# Herpes Zoster Vaccine Uptake in Saudi Arabia: Factors Influencing Adults’ Willingness to Receive the Vaccine

**DOI:** 10.7759/cureus.89824

**Published:** 2025-08-11

**Authors:** Heba Dosh, Murouj Almaghrabi, Jehad Al Qurashi

**Affiliations:** 1 General Practice, Heraa General Hospital, Makkah, SAU; 2 Preventive Medicine, Makkah Health Cluster, Makkah, SAU; 3 Preventive Medicine, Ministry of Health Holdings, Makkah, SAU

**Keywords:** healthcare systems, herpes zoster virus, public health policy, saudi arabia, shingles vaccine

## Abstract

Objective: This study aims to evaluate adults’ willingness to receive the herpes zoster (HZ) vaccine in Saudi Arabia and identify key factors influencing their decisions.

Methods: A cross-sectional study was conducted among adults aged ≥18 years across five Saudi regions from August to September 2024. Data were collected using an Arabic electronic questionnaire covering vaccination prevalence, knowledge, willingness, barriers, and strategies to enhance HZ vaccine uptake.

Results: Among 579 participants, only 14.3% of older adults aged 50+ years (54/377) received the vaccine, with an overall HZ vaccination rate of 10.9%. The primary motivators were healthcare provider recommendations (57.1%) and awareness of benefits (34.9%). Of non-vaccinated individuals, 51.7% expressed willingness to vaccinate if barriers were removed, while 48.3% remained unwilling. The main barrier was a lack of awareness (41.9%). Vaccination rates were significantly higher among residents of the eastern region (26.5%, p = 0.001), those aged 60+ years (25.6%, p = 0.001), and urban residents (12.6%, p = 0.004). Among the non-vaccinated, willingness to vaccinate was higher among individuals from the southern region (64.3%, p = 0.001), aged 30-49 years (53.8%, p = 0.001), urban dwellers (52.3%, p = 0.004), highly educated (56.4%, p = 0.020), recipients of other vaccines (57.7%, p = 0.001), and those with prior shingles diagnoses (57.9%, p = 0.006).

Conclusion: HZ vaccination remains underutilized in Saudi Arabia despite moderate awareness levels. Healthcare provider recommendations, public education, and system-level interventions are critical to improving uptake. National strategies must address disparities in access, knowledge, and provider engagement to enhance preventive healthcare outcomes for at-risk populations.

## Introduction

The varicella-zoster virus (VZV) causes chickenpox, characterized by a maculopapular and vesicular rash that crusts over within three to seven days [1]. Reactivation of the latent virus leads to herpes zoster (HZ), also known as shingles, which manifests as a vesicular rash confined to a dermatome, accompanied by unilateral radicular discomfort linked to the sensory ganglia [1,2]. These symptoms are associated with the sensory ganglion, which reactivates the latent VZV [1,3]. HZ reactivation can result from exposure to active viremia or childhood vaccination, with most adults being seropositive for VZV and at increased risk of HZ [1]. In addition, chronic diseases and aging are major risk factors, as 25% of individuals develop HZ in their lifetime [4-6]. Without vaccination, HZ can result in post-herpetic neuralgia, encephalitis, pneumonia, and ophthalmic complications [7-9].

Preventive measures, especially immunization, are critical in reducing the burden of HZ [10]. Hence, two vaccines are used for varicella and HZ: the live attenuated vaccine (ZOSTAVAX) for individuals aged 60 years and above, and the recombinant subunit vaccine (SHINGRIX) for those aged 50 years and above or 18 years and above with immunocompromising conditions. SHINGRIX, now licensed in Saudi Arabia, is available at all primary care centers [11,12]. However, despite the availability of the vaccine in Saudi Arabia, uptake is remarkably low, posing a significant risk to aging adults by increasing the burden of HZ and its complications [12,13]. In a study conducted in Qassim city, Saudi Arabia, only 25% of diabetic patients were initially willing to accept the HZ vaccine. Interestingly, this figure increased dramatically to 74.2% of the participants who reported their acceptability of vaccination if advised by their physician, underscoring the critical influence of healthcare providers [12]. Another study that has concentrated on particular geographic regions or risk groups indicate that more research is required to comprehend the knowledge and attitudes toward HZ fully and its vaccine in Saudi Arabia, as such efforts would help to decrease the incidence of HZ and its sequelae, which are primarily dependent on vaccination rates, especially among older adults [13].

Despite extensive research efforts in Saudi Arabia, insights are still needed to help health policymakers and practitioners design targeted interventions that maximize vaccination rates and reduce the incidence of HZ within the population. Therefore, this study aims to evaluate adults’ willingness to receive the HZ vaccine in Saudi Arabia and identify key factors influencing their decisions. Furthermore, a predictive model was developed to assess the likelihood of vaccine uptake among the Saudi population.

## Materials and methods

Population and sample

This analytical cross-sectional study was carried out between August and September 2024 and examined adults aged 18 years and above residing in Saudi Arabia. Eligible subjects were individuals who could read Arabic. Individuals with cognitive impairments or those who refused to participate were excluded from the study. We employed a two-stage cluster sampling approach. In stage 1, we randomly selected cities across each of the five principal regions of Saudi Arabia (Central, Western, Eastern, Northern, and Southern). A list of eligible cities was compiled for each region, and three cities were randomly selected using a computer-generated random number sequence. In stage 2, we used convenience sampling to collect data from each selected city. Trained data collectors approached eligible adults using social media platforms. Although convenience sampling limits generalizability, it was chosen as the most feasible method to access a diverse cross-section of adults owing to logistical constraints that precluded the use of a more robust sampling technique. The minimum required sample size was calculated to be 384 participants using OpenEpi software (version 2.1), based on a 95% confidence interval and a 5% margin of error [14].

Ethical considerations

This study obtained ethical approval from the Biomedical Ethics Committee of the Local Research Ethics Committee at the Ministry of Health in the Makkah region (Institutional Review Board reference number: H-02-K-076-0824-1158). All participants provided consent prior to participation. They were assured of confidentiality, voluntary participation, and the right to withdraw at any time.

Instrument validity and reliability

This study employed a structured, self-administered online questionnaire, originally developed by the authors in Arabic. The questionnaire was informed by the thematic domains and item categories presented in a previous study [15]. Substantial modifications were made to enhance its relevance and match the aim of the present study (see Appendix for the complete questionnaire). The questionnaire was adapted to the Arabic language using the forward-backward translation method to ensure linguistic accuracy. Then, it was validated through expert review (face validation) and piloted with 100 participants. The piloted data were used solely to assess feasibility and refine the questionnaire. Internal consistency of the questionnaire was assessed using Cronbach’s alpha, which showed acceptable reliability across the key domains (α = 0.78). These data were excluded from the final analysis. The questionnaire comprised four key sections. The first section collected detailed demographic information, including age, gender, area of residence, educational attainment, and level of income. The second section addressed participants' health status, including their history of chronic conditions, self-reported health status, smoking status, and vaccination history, including whether they received the HZ vaccine or not. The third section assessed participants' knowledge of HZ and its vaccine through 10 structured multiple-choice questions. The final section evaluated participants' willingness to receive the HZ vaccine among non-vaccinated participants, their vaccination behaviors, and perceived barriers to uptake.

Definitions

Smoking status was categorized based on the reported definitions by the Centers for Disease Control and Prevention (CDC) [16], which include the following: (1) every-day smoker (regular smoker): an adult who has smoked at least 100 cigarettes in their lifetime and who now smokes every day; (2) occasional smoker: an adult who has smoked at least 100 cigarettes in their lifetime and who smokes now but does not smoke every day; (3) ex-smoker: an adult who has smoked at least 100 cigarettes in their lifetime and has quit smoking for at least the last 28 days; (4) never smoker: an adult who has never smoked any tobacco products or has smoked fewer than 100 cigarettes in their lifetime and has now stopped.

For self-rated health status, participants were asked to rate their current health status on a 10-point Likert scale, where 1 represented "very poor" health and 10 represented "excellent" health. This approach followed established methods in health research, where self-rated health is frequently used as a validated indicator of overall health status and has shown strong predictive validity for mortality and morbidity outcomes [17]. For the purpose of analysis, self-rated health ratings were divided into three categories: poor (ratings 1-4), average (ratings 5-7), and excellent (ratings 8-10).

Statistical analysis

Descriptive analysis was used for categorical data and reported as frequencies and percentages. Data regarding participants' demographics, vaccination background, knowledge about the HZ vaccine, and their willingness to receive the vaccine were tabulated. While data on reasons for not receiving the HZ vaccine and participants' suggestions to improve willingness to receive the vaccine were graphed. Cross-tabulation was done for factors associated with HZ vaccination. Factors associated with willingness to receive the vaccine among non-vaccinated individuals were analyzed using Pearson’s chi-squared test and the exact probability test for small frequency distributions. No missing data were encountered in the final dataset used for analysis.

Data were analyzed using SPSS version 26 (IBM Corp., Armonk, NY). All statistical methods used were two-tailed with an alpha level of 0.05, and significance was considered if the p-value was less than or equal to 0.05.

## Results

A total of 579 eligible participants completed the study questionnaire. Most participants were from the western region (36.6%), while the eastern region had the lowest representation (11.7%). Over half of the participants were 50-59 years old (50.9%), while younger adults under 30 years of age constituted 21.2%. Males represented 54.4% of the participants. A large proportion (96.9%) were Saudi nationals, and most residents were in urban areas (82.0%).

A significant majority of the participants were married (74.3%), more than half had a college degree (56.5%), and 17.6% had a post-graduate degree, indicating a high level of education in this sample. Employment status varied, with about half being employed (50.4%), and a substantial percentage were retired (26.6%). Only 10.5% of participants worked in health-related occupations, among which physicians represented the largest group (34.4%). The majority reported an income above 10,000 Saudi riyals (SR) per month (57.9%), which is relatively high. A smaller percentage chose not to disclose their income (13.0%). Table 1 provides a comprehensive view of the socio-demographic characteristics and the diversity of the sample.

**Table 1:** Socio-demographic characteristics of study adults in Saudi Arabia (n = 579). SR: Saudi riyal.

Factors		No	%
Region	Western region	212	36.6%
	Southern region	117	20.2%
	Northern region	97	16.8%
	Central region	85	14.7%
	Eastern region	68	11.7%
Age in years	<30	123	21.2%
	30-49	79	13.6%
	50-59	295	50.9%
	60+	82	14.2%
Gender	Male	315	54.4%
	Female	264	45.6%
Nationality	Saudi	561	96.9%
	Non-Saudi	18	3.1%
Residence	Rural area	104	18.0%
	Urban area	475	82.0%
Marital status	Single	117	20.2%
	Married	430	74.3%
	Divorced/widow	32	5.5%
Completed educational level	None/basic education	30	5.2%
	High school	120	20.7%
	College degree	327	56.5%
	Post-graduate degree	102	17.6%
Employment status	Employed	292	50.4%
	Unemployed	133	23.0%
	Retired	154	26.6%
Do you work in a health-related occupation?	Yes	61	10.5%
	No	518	89.5%
If you work in a health-related occupation, please specify the job title	Physician	21	34.4%
	Not mentioned	16	26.2%
	Nursing	9	14.8%
	Paramedical	6	9.8%
	Laboratory	4	6.6%
	Dentist	1	1.6%
	Dentistry	1	1.6%
	Nurse	1	1.6%
	Pharmacist	1	1.6%
	Radiology	1	1.6%
Monthly income	<5000 SR	38	6.6%
	5000-10000 SR	131	22.6%
	> 10000 SR	335	57.9%
	Prefer not to tell	75	13.0%

The participants’ medical and health status are shown in Table 2. Most participants rated their health status positively, with 72.0% considering it excellent (8-10), 23.0% rating it as average (5-7), and only 5.0% reporting it as poor (1-4). Regarding smoking status, 72.0% of participants had never smoked, while 12.6% smoked daily, 6.6% smoked occasionally, and 8.8% were ex-smokers. The majority (69.1%) had no chronic disease. Among those with health concerns, diabetes mellitus (18.7%) was the most commonly reported condition, followed by rheumatoid arthritis (6.2%) and cardiovascular disease (5.5%). Other health issues were less prevalent, with kidney diseases affecting 1.9%, inflammatory bowel disease affecting 1.2%, and cancer and immunosuppressive treatment each affecting 0.9%.

**Table 2:** Health status characteristics of study participants in Saudi Arabia (n = 579). ^§^ As this was a multiple-choice question, percentages represent the proportion of participants who selected each option, rather than totaling 100%.

Medical data	No.	%
Self-rating of the participant's current health status		
Poor (1-4)	29	5.0%
Average (5-7)	133	23.0%
Excellent (8-10)	417	72.0%
Smoking status		
Smoke daily	73	12.6%
Smoke sometimes	38	6.6%
Ex-smoker	51	8.8%
Never smoked	417	72.0%
Chronic health problems^§^		
No known medical illness	400	69.1%
Diabetes mellitus	108	18.7%
Rheumatoid arthritis	36	6.2%
Cardiovascular disease	32	5.5%
Depression	14	2.4%
Kidney diseases	11	1.9%
Inflammatory bowel disease (Crohn's disease or ulcerative colitis)	7	1.2%
Cancer	5	0.9%
Taking any immunosuppressive medications (such as steroids or chemotherapy)	5	0.9%
Lupus erythematosus	3	0.5%
Others	55	9.5%
Have you ever been diagnosed with chickenpox?		
Yes	115	19.9%
No	382	66.0%
I do not know - not sure	82	14.2%
Have you ever been diagnosed with shingles (herpes zoster)?		
Yes	27	4.7%
No	525	90.7%
I do not know - not sure	27	4.7%
Have you ever received immunization vaccines (like seasonal influenza and pneumococcal vaccines)?		
Yes	334	57.7%
No	245	42.3%
Do you usually refuse/or have you previously refused to receive any vaccine/s?		
Yes	71	12.3%
No	506	87.7%
If yes, which vaccines? (n = 71)		
Flu vaccine	20	28.2%
COVID vaccine	15	21.1%
Shingles vaccine	10	14.1%
Others/do not remember the vaccine’s name	26	36.6%

A minority of the participants had been previously diagnosed with chickenpox (19.9%), and 14.2% were unsure. Furthermore, 27 (4.7%) had been previously diagnosed with HZ. More than half (57.7%, n = 334) of the participants received immunization vaccines (such as seasonal influenza and pneumococcal vaccines), while 71 (12.3%) usually refused to or had previously refused to receive any vaccines.

Table 3 shows the knowledge and awareness of HZ and its vaccine among adults in Saudi Arabia. Out of the 579 participants, a majority (71.7%) had heard of HZ. When asked whether having had chickenpox increases the risk of HZ, only 17.8% answered correctly, 8.8% were incorrect, and most of them (73.4%) were unsure. Furthermore, 40.6% answered correctly about the risks related to HZ for people with immunodeficiency, while 7.1% were incorrect, and 52.3% did not know. Only 13.0% knew that teens could contract HZ, while 25.9% misbelieved that they could not, and 61.1% did not know.

**Table 3:** Knowledge and awareness about herpes zoster and its vaccine in Saudi Arabia (n = 579).

Knowledge		No.	%
Have you ever heard of herpes zoster?	Yes	415	71.7%
	No	164	28.3%
If a person has previously been exposed to chicken pox, this will increase their risk of developing herpes zoster	Correct	103	17.8%
	Incorrect	51	8.8%
	I do not know	425	73.4%
People with immunodeficiency are more susceptible to herpes zoster	Correct	235	40.6%
	Incorrect	41	7.1%
	I do not know	303	52.3%
People in their teens will never get herpes zoster	Correct	75	13.0%
	Incorrect	150	25.9%
	I do not know	354	61.1%
Contacting people with herpes zoster leads to direct infection with the disease	Correct	100	17.3%
	Incorrect	129	22.3%
	I do not know	350	60.4%
There are no medications available to treat herpes zoster	Correct	47	8.1%
	Incorrect	239	41.3%
	I do not know	293	50.6%
Symptoms of herpes zoster	Skin rash	279	48.2%
	Skin blisters	279	48.2%
	Neuropathic pain	567	97.9%
	Hearing loss	19	3.3%
	Death	26	4.5%
	Loss of vision	20	3.5%
The herpes zoster vaccine can reduce the incidence of the disease by more than 50%	Correct	268	46.3%
	Incorrect	19	3.3%
	I do not know	292	50.4%
The herpes zoster vaccine can treat an active infection	Correct	117	20.2%
	Incorrect	66	11.4%
	I do not know	396	68.4%
Which age group has been recommended to receive the herpes zoster vaccine?	Any age group can receive the vaccine	50	8.6%
	18 years or older	39	6.7%
	50 years or more	277	47.8%
	I do not know	213	36.8%
Which group of people has been recommended to take the herpes zoster vaccine?	He has not been infected with or is not sure whether he has chickenpox	83	14.3%
	He had previously had chickenpox, but not herpes zoster	85	14.7%
	He had herpes zoster previously	26	4.5%
	I do not know	385	66.5%
The most common source of information about vaccines	None	49	9.5%
	Internet or social media	221	42.8%
	Healthcare professionals	171	33.1%
	Family and friends	46	8.9%
	Television/radio	29	5.6%

Regarding whether HZ is contagious through direct contact, 17.3% answered correctly, but 22.3% were mistaken, and 60.4% were unsure. Regarding treatment, only 8.1% knew that medications were available for HZ, while 41.3% incorrectly believed there were none, and 50.6% were uncertain. Regarding symptoms, 48.2% identified skin rash and blisters, while 97.9% were aware of neuropathic pain, and fewer reported less common outcomes, such as hearing loss (3.3%), death (4.5%), or vision loss (3.5%). As for the vaccine, 46.3% knew that it reduces the incidence of HZ by over 50%, 3.3% were incorrect, and 50.4% did not know. Only 20.2% understood that the vaccine does not treat active infection (11.4% were wrong, and 68.4% were uncertain).

In regard to the recommended age for vaccination, 47.8% correctly cited 50 years or older, while others thought it applied to any age (8.6%) or those 18 years and older (6.7%), and 36.8% were unsure. When asked who should receive the vaccine, 14.3% correctly identified those who are uncertain whether they had chickenpox or were never infected, 14.7% noted those with previous chickenpox infection but not HZ, 4.5% thought it was for those who previously had HZ, and 66.5% did not know. The most common source of information about vaccines was the internet or social media (42.8%), followed by healthcare professionals (33.1%), and family and friends (8.9%), while 9.5% had no specific source.

Table 4 provides insights into the willingness and factors influencing the decision to receive the HZ vaccine among adults in Saudi Arabia. Among older adults aged 50 years and above, only 14.3% (54 out of 377 participants) had received the HZ vaccine. The total prevalence of HZ vaccination across all age groups was 10.9% (n = 63), while a substantial majority (89.1%, n = 516) had not received it. Among those who were vaccinated (n = 63), healthcare provider recommendations were the most significant motivator, with 57.1% citing this as their primary reason for vaccination. Additionally, 34.9% of vaccinated respondents recognized the benefits of the vaccine, and 7.9% decided to receive it due to a personal or family history of HZ.

**Table 4:** Willingness to receive the herpes zoster vaccine in Saudi Arabia (n = 579).

Willingness	No.	%
Prevalence of older adults who received the herpes zoster vaccine (n = 377)		
Received the vaccine	54	14.3%
Did not receive the vaccine	323	85.7%
Total prevalence of adults who received the herpes zoster vaccine (across all participants, n = 579)		
Received the vaccine	63	10.9%
Did not receive the vaccine	516	89.1%
If received, what were the main reasons for your decision? (n = 63)		
Healthcare provider's recommendation	36	57.1%
Personal or family history of shingles	5	7.9%
Awareness of vaccine benefits	22	34.9%
If the vaccine were available with no barriers, would you agree to receive it? (n = 516)		
Yes	267	51.7%
No	249	48.3%
Has your healthcare provider recommended the herpes zoster vaccine to you? (n = 516)		
Yes	64	12.4%
No	210	40.7%
Don't have a healthcare provider	242	46.9%
Would a recommendation from a healthcare provider influence your decision and let you take the vaccine? (n = 516)		
Yes	297	57.6%
No	219	42.4%
How likely are you to get the vaccine if a close friend or family member recommends it? (n = 516)		
Very likely	149	28.9%
Somewhat likely	193	37.4%
Not likely	99	19.2%
Very unlikely	75	14.5%
How much does the opinion of your family influence your decision to get vaccinated? (n = 516)		
A lot	146	28.3%
A little	186	36.0%
Not at all	184	35.7%
Have you ever changed your mind about getting vaccinated because of someone else’s opinion or experience? (n = 516)		
Yes	172	33.3%
No	344	66.7%
Are there any specific cultural or religious concerns in your community that affect decisions about vaccination? (n = 516)		
Yes	51	9.9%
No	465	90.1%
Have you ever wanted to get a vaccine but were unable to? (n = 516)		
Yes	25	4.8%
No	491	95.2%
If yes, what were the reasons? (n = 25)		
Unavailable	16	64.0%
No reason	4	16.0%
Don't know place to get the vaccine	1	4.0%
Lack of awareness	1	4.0%
Laziness	1	4.0%
Time schedule	1	4.0%
Young age	1	4.0%

Among the non-vaccinated, 51.7% (n = 516) expressed a willingness to be vaccinated if it were available without barriers, while 48.3% remained unwilling. Of those who had not yet received the vaccine, only 12.4% indicated they had received a recommendation from a healthcare provider to be vaccinated, while 46.9% reported not having a healthcare provider at all, suggesting a gap in healthcare access or engagement. A recommendation from a healthcare provider could potentially influence a significant portion, with 57.6% stating it would make them more likely to accept the vaccine. Also, when asked about the likelihood of being vaccinated if encouraged by family or friends, 28.9% said they would be "very likely" to receive the vaccine, while 37.4% responded they would be "somewhat likely." For 28.3%, family opinions significantly influenced their vaccination decision, and an additional 36.0% reported moderate influence from family.

Cultural or religious concerns did not appear to be major barriers, as 90.1% stated there were no such concerns in their community regarding vaccination. However, 9.9% acknowledged specific cultural or religious issues that could affect vaccine decisions. Barriers to vaccination were infrequent, with only 4.8% of respondents reporting that they had wanted to receive a vaccine but were unable to. Among this small group (n = 25), unavailability was the most common reason (64%), followed by a variety of lesser factors like scheduling conflicts, lack of awareness, and the belief that they were too young, each accounting for 4% of the responses.

**Figure 1:**
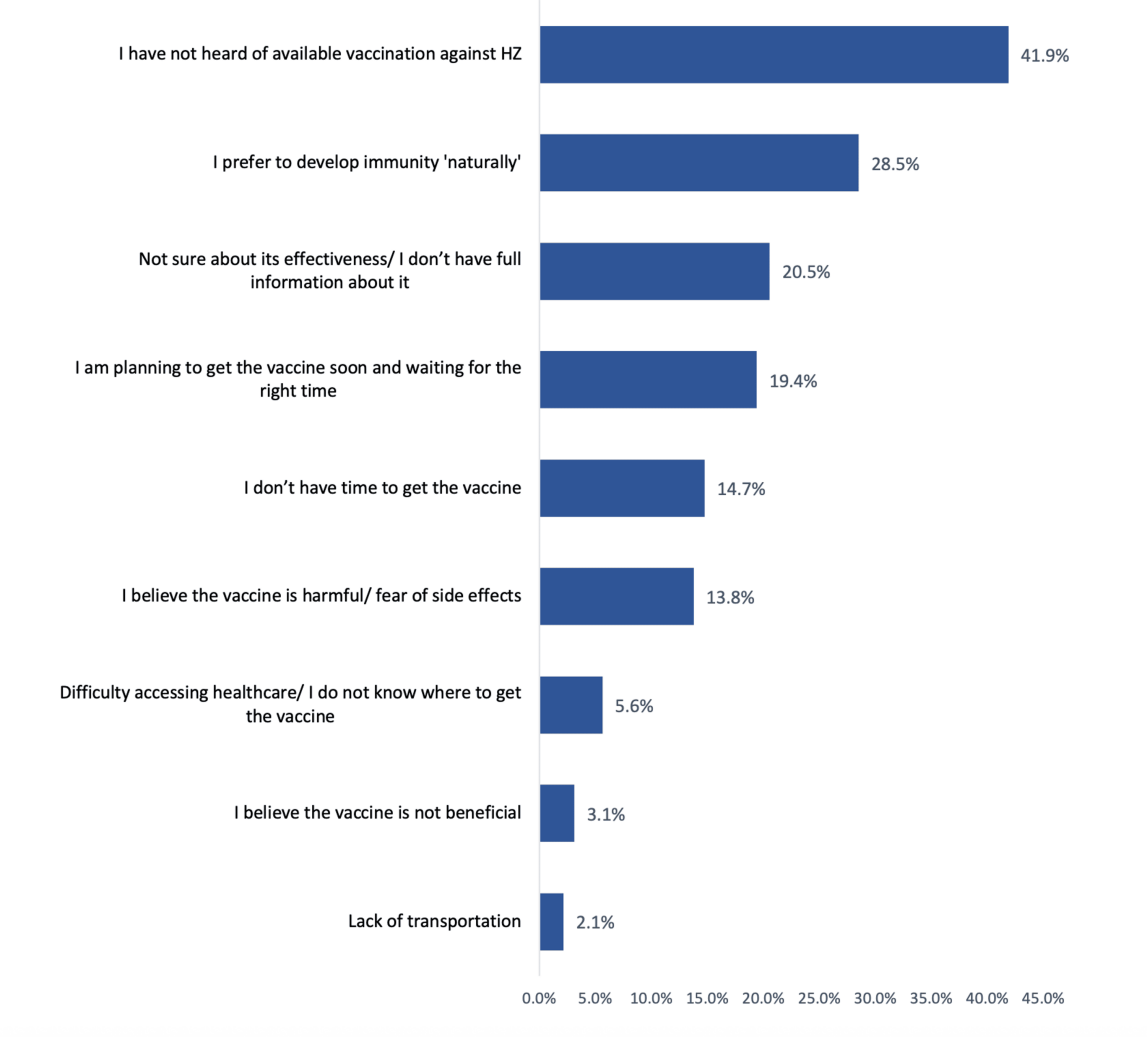
The most reported reasons for not receiving the herpes zoster vaccine among study adults in Saudi Arabia (n = 516). HZ: herpes zoster.

The most reported reasons for not receiving the HZ vaccine among adults in Saudi Arabia were lack of awareness about available vaccination against HZ (41.9%), preference to develop immunity “naturally” (28.5%), not being sure about its effectiveness/not having full information about it (20.5%), planning to receive the vaccine soon and waiting for the right time (19.4%), not having time to receive the vaccine (14.7%), and belief that the vaccine is harmful or fear of side effects (13.8%) (Figure 1).

**Figure 2:**
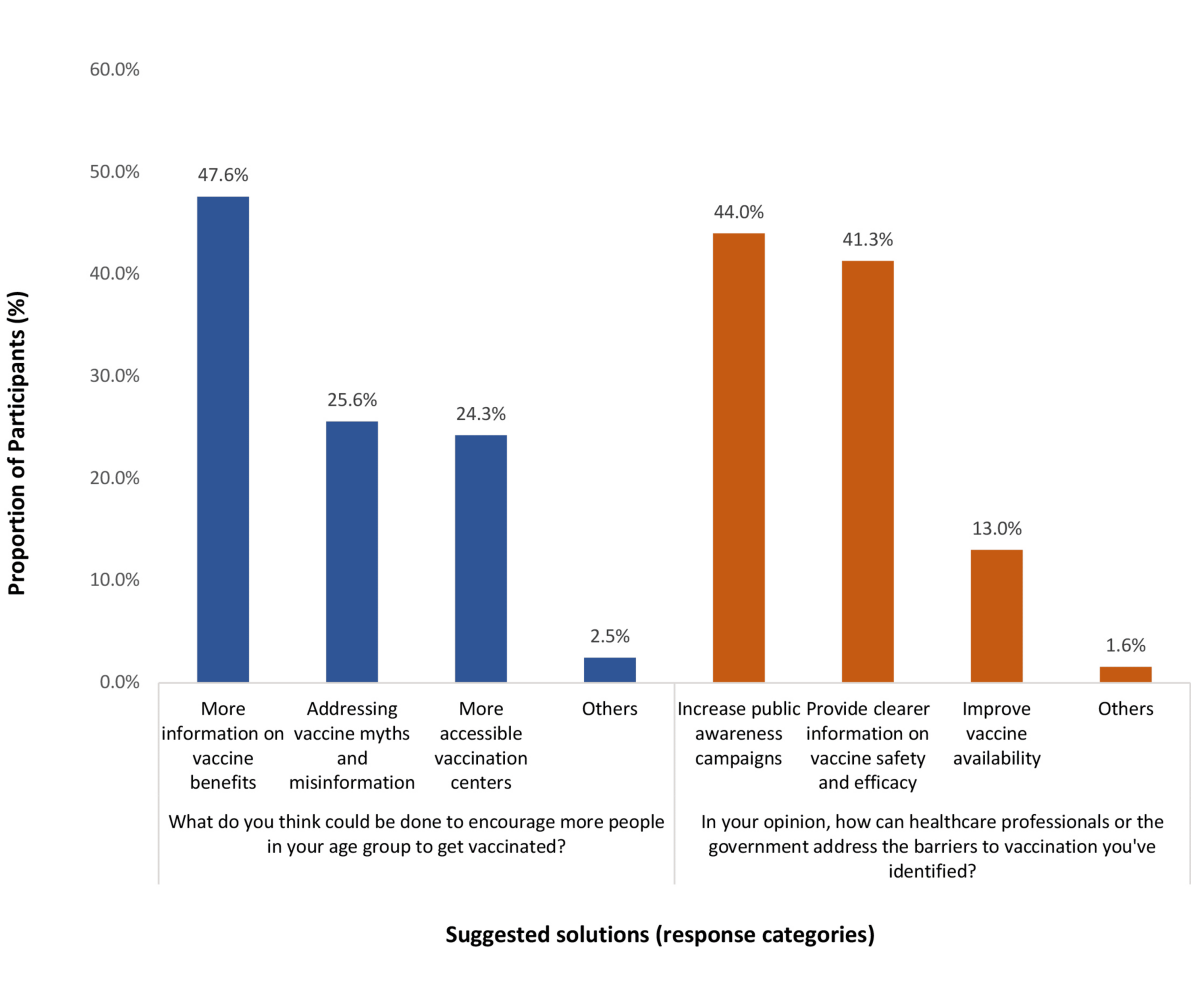
Participants' suggested solutions to improve willingness for the herpes zoster vaccine in Saudi Arabia.

The most reported suggestions to encourage more people in one’s age group to be vaccinated included providing more information about vaccine benefits (47.6%), addressing vaccine myths and misinformation (25.6%), and having more accessible vaccination centers (24.3%). The most reported suggestions for healthcare professionals or the government to address the barriers to vaccination were increasing public awareness campaigns (44%), providing clearer information on vaccine safety and efficacy (41.3%), and improving vaccine availability (13%) (Figure 2).

Table 5 shows the factors associated with HZ vaccination among the participants. Those from the eastern region had a higher vaccination rate (26.5%) compared to other regions, and the lowest was in the southern region (4.3%) (p = 0.001). Vaccination rates increased with age, especially among adults aged 60+ years (25.6%). The lowest rate was among those aged 30-49 years (1.3%) (p = 0.001).

**Table 5:** Factors associated with herpes zoster vaccination among the study adults. χ² = Pearson’s chi-square statistic. ^ Exact probability test. * P < 0.05 (significant).

Factors		Status of herpes zoster vaccine				p-value
		Received		Not received		
		No.	%	No.	%	
Region	Central region	9	10.6%	76	89.4%	0.001*
	Eastern region	18	26.5%	50	73.5%	
	Northern region	8	8.2%	89	91.8%	
	Southern region	5	4.3%	112	95.7%	
	Western region	23	10.8%	189	89.2%	
Age in years	<30	8	6.5%	115	93.5%	0.001*
	30-49	1	1.3%	78	98.7%	
	50-59	33	11.2%	262	88.8%	
	60+	21	25.6%	61	74.4%	
Gender	Male	34	10.8%	281	89.2%	0.941
	Female	29	11.0%	235	89.0%	
Nationality	Saudi	61	10.9%	500	89.1%	0.975^
	Non-Saudi	2	11.1%	16	88.9%	
Residence	Rural area	3	2.9%	101	97.1%	0.004*
	Urban area	60	12.6%	415	87.4%	
Completed educational level	None/basic education	4	13.3%	26	86.7%	0.020*
	High school	19	15.8%	101	84.2%	
	College degree	24	7.3%	303	92.7%	
	Post-graduate degree	16	15.7%	86	84.3%	
Do you work in a health-related occupation?	Yes	5	8.2%	56	91.8%	0.477
	No	58	11.2%	460	88.8%	
Rate your current health status	Poor (1-4)	2	6.9%	27	93.1%	0.379
	Average (5-7)	11	8.3%	122	91.7%	
	Excellent (8-10)	50	12.0%	367	88.0%	
Smoking status	Smoke daily	5	6.8%	68	93.2%	0.006*
	Smoke sometimes	1	2.6%	37	97.4%	
	Ex-smoker	12	23.5%	39	76.5%	
	Never smoked	45	10.8%	372	89.2%	
Have you ever been diagnosed with chickenpox?	Yes	7	6.1%	108	93.9%	0.088
	No	43	11.3%	339	88.7%	
	I do not know - not sure	13	15.9%	69	84.1%	
Have you ever received immunization vaccines (like seasonal influenza and pneumococcal vaccines)?	Yes	50	15.0%	284	85.0%	0.001*
	No	13	5.3%	232	94.7%	
Have you ever heard of shingles (herpes zoster)?	Yes	58	14.0%	357	86.0%	0.001*
	No	5	3.0%	159	97.0%	
Have you ever been diagnosed with shingles (herpes zoster)?	Yes	8	29.6%	19	70.4%	0.006*^
	No	52	9.9%	473	90.1%	
	I do not know - not sure	3	11.1%	24	88.9%	

As for residence, adults living in urban areas had a significantly higher vaccination rate (12.6%) compared to rural residents (2.9%) (p = 0.004). Participants with high-school education (15.8%) and post-graduate degrees (15.7%) reported higher vaccination rates than those with a college degree (7.3%) (p = 0.020). Regarding smoking status, ex-smokers showed the highest vaccination rate (23.5%), while those who smoked sometimes had the lowest (2.6%) (p = 0.006). Individuals who had previously received immunization vaccines had a higher HZ vaccination rate (15.0%) compared to those who had not (5.3%) (p = 0.001). Additionally, adults who had heard of HZ reported a higher vaccination rate (14.0%) than those who had not (3.0%) (p = 0.001), and similarly, those previously diagnosed with HZ had a significantly higher vaccination rate (29.6%) compared to those without a diagnosis (9.9%) (p = 0.006).

Table 6 shows the factors associated with willingness to receive the HZ vaccine among non-vaccinated adults. In the southern region, 64.3% of participants indicated willingness to receive the vaccine, which was significantly higher than in other regions (p = 0.001). Also, participants aged 30-49 were 53.8% willing, while only 38.3% of those under 30 years old (p = 0.001). Urban residents had a higher willingness (52.3%) compared to rural residents (49.5%) (p = 0.004), while 56.4% of participants with a high-school education reported willingness, and those with basic education had the lowest willingness (38.5%) (p = 0.020). Regarding smoking status, ex-smokers had the highest willingness at 64.1%, while those who smoked daily were 47.1% willing (p = 0.006).

**Table 6:** Factors associated with herpes zoster vaccination willingness among the study non-vaccinated adults. ^§^ To assess the non-vaccinated participants' willingness to receive herpes zoster vaccination, we asked the following question: "If the vaccine were available with no barriers, would you agree to receive it?" χ² = Pearson’s chi-square statistic. ^ Exact probability test. * P < 0.05 (significant).

Factors		Willing to receive the vaccine^§^				p-value
		Yes		No		
		No.	%	No.	%	
Region	Central region	38	50.0%	38	50.0%	0.001*
	Eastern region	25	50.0%	25	50.0%	
	Northern region	42	47.2%	47	52.8%	
	Southern region	72	64.3%	40	35.7%	
	Western region	90	47.6%	99	52.4%	
Age in years	<30	44	38.3%	71	61.7%	0.001*
	30-49	42	53.8%	36	46.2%	
	50-59	150	57.3%	112	42.7%	
	60+	31	50.8%	30	49.2%	
Gender	Male	146	52.0%	135	48.0%	0.941
	Female	121	51.5%	114	48.5%	
Nationality	Saudi	258	51.6%	242	48.4%	0.975^
	Non-Saudi	9	56.3%	7	43.8%	
Residence	Rural area	50	49.5%	51	50.5%	0.004*
	Urban area	217	52.3%	198	47.7%	
Completed educational level	None/basic education	10	38.5%	16	61.5%	0.020*
	High school	57	56.4%	44	43.6%	
	College degree	161	53.1%	142	46.9%	
	Post-graduate degree	39	45.3%	47	54.7%	
Do you work in a health-related occupation?	Yes	29	51.8%	27	48.2%	0.477
	No	238	51.7%	222	48.3%	
Rate your current health status	Poor (1-4)	13	48.1%	14	51.9%	0.379
	Average (5-7)	64	52.5%	58	47.5%	
	Excellent (8-10)	190	51.8%	177	48.2%	
Smoking status	Smoke daily	32	47.1%	36	52.9%	0.006*
	Smoke sometimes	19	51.4%	18	48.6%	
	Ex-smoker	25	64.1%	14	35.9%	
	Never smoked	191	51.3%	181	48.7%	
Have you ever been diagnosed with chickenpox?	Yes	60	55.6%	48	44.4%	0.088
	No	178	52.5%	161	47.5%	
	I do not know - not sure	29	42.0%	40	58.0%	
Have you ever received immunization vaccines (like seasonal influenza and pneumococcal vaccines)?	Yes	164	57.7%	120	42.3%	0.001*
	No	103	44.4%	129	55.6%	
Have you ever heard of shingles (herpes zoster)?	Yes	201	56.3%	156	43.7%	0.001*
	No	66	41.5%	93	58.5%	
Have you ever been diagnosed with shingles (herpes zoster)?	Yes	11	57.9%	8	42.1%	0.006*^
	No	244	51.6%	229	48.4%	
	I do not know - not sure	12	50.0%	12	50.0%	

Willingness to receive the vaccine was significantly higher among individuals who had received other immunization vaccines (57.7%) compared to those who had not (44.4%) (p = 0.001). Regarding awareness, adults who had heard of HZ demonstrated a higher willingness (56.3%) than those who had not (41.5%) (p = 0.001). Additionally, 57.9% of participants who had previously been diagnosed with HZ showed willingness, while 51.6% of those without a diagnosis were willing (p = 0.006).

## Discussion

HZ represents a significant health burden, particularly for older adults. This study assessed adults’ willingness to receive the HZ vaccine in Saudi Arabia, identifying key factors that influence their decisions. Over half the participants (50.9%) were aged 50-59 years, a critical group for HZ vaccination, as recommended by the CDC [18]. A majority (56.5%) held a college degree, a factor positively associated with vaccine awareness and acceptance [19]. However, only 10.5% were healthcare professionals, with physicians comprising the largest subgroup (34.4%). The influence of healthcare professionals on vaccine uptake highlights the need to engage non-healthcare workers in vaccination awareness efforts, thereby extending outreach beyond healthcare settings [20].

A notable 72% of participants rated their health as excellent, a significantly higher rate than typically reported in the region, where older adults often report moderate or poor health [21]. However, the prevalence of chronic conditions such as diabetes mellitus (18.7%) aligns with national trends linked to lifestyle factors [22]. Smoking rates were low, with 72% identifying themselves as never smokers, a trend reflecting healthier behaviors and potentially greater openness to preventive health measures, such as vaccination [23]. Furthermore, 4.7% reported a history of HZ, which aligns with epidemiological estimates of HZ prevalence; however, it may also indicate underreporting due to limited awareness or misdiagnosis [24].

Despite a high proportion of participants who had heard of HZ (71.7%), only 17.8% of participants understood the link between chickenpox and the risk of HZ, while 73.4% were unaware of this connection. Misconceptions persisted, with 60.4% unsure whether contact with HZ patients could lead to direct infection and 50.6% unaware of available treatments. These findings align with global trends, where awareness often fails to translate into a deeper understanding [25,26]. Vaccine awareness about its efficacy and eligibility was moderate; while 46.3% recognized its effectiveness in reducing HZ incidence by over 50%, 50.4% were unaware of this benefit. These findings align with a randomized controlled trial conducted in the United Kingdom, where vaccine knowledge was found to be insufficient even among older adults [27]. Notably, 66.5% of participants were unaware of the criteria for receiving the vaccine, and misconceptions persisted about eligibility based on prior infection history. This finding is consistent with a study by Al-Dahshan et al. in Qatar, which also highlighted confusion regarding vaccine eligibility among adults [28].

The vaccination rate for HZ in Saudi Arabia remains low. In this study, 10.9% of participants reported receiving the vaccine, and only 14.3% of older adults were vaccinated. This aligns with regional data from a recent study in Saudi Arabia, which found that less than 7.7% of individuals had been vaccinated against HZ [13]. However, these rates are significantly lower than those reported in high-income countries such as the United States, where vaccination rates reach 34% [29]. In contrast, rates in Europe and Asia remain below 20% [26]. Barriers to vaccination included lack of healthcare provider recommendations (41.9%), insufficient knowledge (28.5%), and unawareness of vaccine availability (20.5%). These barriers highlight the crucial role of healthcare professionals, as their recommendations are strongly correlated with increased vaccine uptake [30]. Interestingly, 51.7% of non-vaccinated participants expressed willingness to vaccinate if barriers were removed, demonstrating the potential for substantial improvements through targeted interventions. Participants from the southern region (64.3%) and those aged 50-59 years (57.3%) exhibited the highest willingness, consistent with global patterns showing higher vaccine acceptance among older adults and those in regions with focused healthcare access [31,32]. Awareness was a pivotal factor; participants familiar with HZ demonstrated greater willingness to vaccinate (56.3%), consistent with findings that knowledge about preventable diseases enhances vaccine acceptance [30].

Healthcare provider recommendations emerged as the most significant motivator for vaccination. According to Hurley et al., provider endorsement is among the strongest predictors of vaccine uptake [33]. Enhancing provider engagement by integrating vaccine discussions into routine consultations and equipping them with up-to-date resources can significantly improve vaccination rates [34]. Public health campaigns should also address persistent misconceptions and knowledge gaps. Comprehensive initiatives leveraging diverse platforms, such as social media, community outreach, and partnerships with healthcare professionals, are essential to improve awareness and accessibility [32,34].

Limited vaccine accessibility, cited by 19.4% of participants, remains a significant barrier to vaccination. Expanding vaccination centers to underserved areas, ensuring consistent supply chains, and incorporating vaccines into primary healthcare facilities could effectively address logistical challenges. These strategies align with recommendations from Wang et al., who emphasize the importance of streamlining vaccination processes in low- and middle-income countries [31].

Study limitations and recommendations

This study provides robust insights into socio-demographic, behavioral, and health-related factors influencing HZ vaccine uptake in Saudi Arabia. Its strengths include a diverse sample of 579 participants from five regions and the use of a culturally adapted, validated questionnaire. These findings contribute valuable evidence for tailoring public health strategies to Saudi Arabia’s healthcare landscape. However, certain limitations must be considered. (1) Regional representation was uneven, with overrepresentation of the western region. (2) The reliance on self-reported data introduces potential inaccuracies, such as recall or social desirability bias, particularly in the context of vaccination history. (3) The cross-sectional design limits causal inferences. (4) As stage two of the sampling strategy relied on convenience sampling, it is likely to over-represent adults with internet access and digital literacy. As a result, our findings may not generalize to individuals without reliable online connectivity. Future studies should aim for balanced regional sampling, normal distribution of age groups, conducting multivariate analysis, employing probability-based household sampling, and longitudinal designs to track changes in attitudes and behaviors, as well as the inclusion of underserved populations.

To improve HZ vaccine uptake, public health initiatives should prioritize integrating vaccine discussions into routine healthcare visits and expanding access to vaccination services. Addressing misinformation through targeted educational campaigns and leveraging the influence of healthcare providers can also drive significant improvements. Future research should focus on interventional studies to evaluate the impact of academic programs and provider training on vaccine uptake, as well as explore systemic barriers, such as cost and availability, that hinder access to vaccines.

## Conclusions

Despite moderate awareness of HZ, the vaccine uptake remains low in Saudi Arabia. Remarkable barriers include regional disparities, age, education, prior health behaviors, knowledge gaps, and a lack of awareness. However, recommendations from healthcare providers and increased public information have enhanced vaccination willingness and uptake rates. These findings underscore the need for targeted public health strategies to improve vaccination rates in each region of Saudi Arabia. Prioritizing educational campaigns, enhancing healthcare provider involvement, and addressing logistical challenges are essential steps toward increasing vaccine acceptance. By addressing these barriers, public health initiatives can reduce the burden of HZ and its complications, ultimately improving the health outcomes of at-risk populations nationwide.

## Figures and Tables

**Figure 3:**
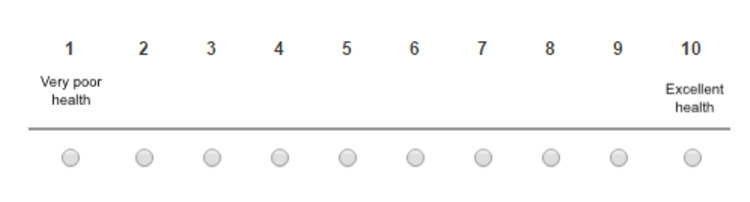
Health status scale.

## Appendices

Questionnaire

1: Demographic and Socio-Economic Characteristics of the Participants

Age: ……………………..

Gender:

(1) Male

(2) Female

Nationality:

(1) Saudi

(2) Non-Saudi

Place of residency:

(1) Central region of Saudi Arabia

(2) Western region of Saudi Arabia

(3) Eastern region of Saudi Arabia

(4) Northern region of Saudi Arabia

(5) Southern region of Saudi Arabia

(6) Out of Saudi Arabia

Area of residency:

(1) Rural area

(2) Urban area

Marital status:

(1) Married

(2) Single

(3) Widowed/divorced

Educational level (The highest level of education successfully completed):

(1) No formal school

(2) Elementary and middle school

(3) High school

(4) College degree

(5) Post-graduate degree

Employment status:

(1) Employed

(2) Unemployed

(3) Retired

Do you work in a health-related occupation?

(1) Yes, specify:……………………………

(2) No

Income level for the family:

(1) Less than 5,000 SAR

(2) 5,000 - 10,000 SAR

(3) More than 10,000 SAR

(4) No Income

(5) I prefer not to tell

2: Participants’ Health Status

On a scale from 1 to 10, with 1 being "very poor" and 10 being "excellent," how would you rate your current health status?

Smoking status: for any kind of tobacco smoking

(1) I smoke daily

(2) I smoke sometimes

(3) Ex-smoker (not smoked at least for the last 28 days)

(4) Never smoked

Do you have any of the following conditions? (Select all that apply)

(1) Cardiovascular disease

(2) Diabetes

(3) Lupus erythematosus

(4) Rheumatoid arthritis

(5) Kidney disease

(6) Inflammatory bowel disease (Crohn's disease or ulcerative colitis)

(7) Cancer

(8) Depression

(9) Taking any immunosuppressive medications (such as steroids or chemotherapy)

(10) I do not have any complaints from the previous list

Do you have any other chronic illnesses or any previous diseases (like stroke)?

(1) Yes, specify:…………………………..

(2) No, I do not have any chronic illnesses.

Have you ever been diagnosed with chickenpox?

(1) Yes

(2) No

(3) I don’t know/ not sure

Have you ever received immunization vaccines (like seasonal influenza and pneumococcal vaccines)?

(1) Yes

(2) No

Have you ever heard of shingles (herpes zoster)?

(1) Yes

(2) No

Have you ever been diagnosed with shingles (herpes zoster)?

(1) Yes

(2) No

(3) I don’t know/not sure

Do you usually refuse/or have you previously refused to receive any of the vaccine/s?

(1) Yes, specify the vaccine/s you refused:………………………………

(2) No

3: Knowledge of Herpes Zoster and Its Vaccination

1: If a person has previously been exposed to chicken pox, this will increase their risk of developing herpes zoster.

- Correct

- Incorrect

- I don't know

2: People with immunodeficiency are more susceptible to herpes zoster.

- Correct

- Incorrect

- I don't know

3: People in their teens will never get herpes zoster.

- Correct

- Incorrect

- I don't know

4: Contacting people with herpes zoster leads to direct infection with the disease.

- Correct

- Incorrect

- I don't know

5: There are no medications available to treat herpes zoster.

- Correct

- Incorrect

- I don't know

6: The following is/are symptom/s of herpes zoster.

- Skin rashes, skin blisters, and neuropathic pain

- Loss of sight

- Hearing loss

- Death

- I don't know

7: The herpes zoster vaccine can reduce the incidence of the disease by more than 50%.

- Correct

- Incorrect

- I don't know

8: Herpes zoster vaccine can treat active infection.

- Incorrect

- Mistake

- I don't know

9: Which age group has been recommended to receive the herpes zoster vaccine?

- Any group can receive the vaccine

- 18 years or older

- 50 years or more

- I don't know

10: Which group of people has been recommended to take the herpes zoster vaccine?

- He has not been infected with or is not sure whether he has chickenpox

- He had previously had chickenpox, but not herpes zoster

- He had herpes zoster previously

- I don't know

4: Herpes Zoster Vaccine Willingness, Behaviors, and Barriers

Have you received the vaccine for herpes zoster?

(1) Yes

(2) No

If yes, what were the main reasons for your decision? (Select all that apply)

(1) Healthcare provider's recommendation

(2) Personal or family history of shingles

(3) Awareness of vaccine benefits

(4) Others, specify:…………………………………………………..

If Yes -> Go to the thank you page.

If No -> Go to the next question:

Why have you not received the vaccine for herpes zoster? (Select all that apply)

(1) I have not heard of an available vaccination against HZ

(2) Difficulty accessing healthcare/I do not know where to get the vaccine

(3) Lack of transportation

(4) Not sure about its effectiveness/I don’t have full information about it

(5) I believe the vaccine is not beneficial

(6) I believe the vaccine is harmful/fear of side effects

(7) I prefer to develop immunity "naturally"

(8) I don’t have time to get the vaccine

(9) I am planning to get the vaccine soon and am waiting for the right time

(10) Others, specify:…………………………………………………..

If the vaccine were available with no barriers, would you agree to receive it? (Directly assessing willingness among non-vaccinated)

(1) Yes

(2) No

Has your healthcare provider recommended the herpes zoster vaccine to you?

(1) Yes

(2) No

(3) Don't have a healthcare provider

Would a recommendation from a healthcare provider influence your decision and let you take the vaccine?

(1) Yes

(2) No

How likely are you to get the vaccine if a close friend or family member recommends it?

(1) Very likely

(2) Somewhat likely

(3) Not likely

(4) Very unlikely

How much does the opinion of your family influence your decision to get vaccinated?

(1) A lot

(2) A little

(3) Not at all

Have you ever changed your mind about getting vaccinated because of someone else’s opinion or experience? Please describe the situation.

(1) Yes, specify:……………………………………………….

(2) No

Are there any specific cultural or religious concerns in your community that affect decisions about vaccination? Please explain.

(1) Yes, specify:……………………………………………….

(2) No

What is the most common source you use to get information about vaccines?

(1) Healthcare professionals

(2) Internet or social media

(3) Family and friends

(4) TV/radio

(5) Other. Specify:……………………………

Have you ever wanted to get a vaccine but were unable to? If yes, what were the reasons?

(1) Yes, specify:……………………………………………….

(2) No

What do you think could be done to encourage more people in your age group to get vaccinated?

(1) More accessible vaccination centers

(2) More information on vaccine benefits

(3) Addressing vaccine myths and misinformation

(4) Other. Specify:……………………………………………

In your opinion, how can healthcare professionals or the government address the barriers to vaccination you've identified?

(1) Improve vaccine availability

(2) Increase public awareness campaigns

(3) Provide clearer information on vaccine safety and efficacy

(4) Others. Specify:……………………………………………

Note: An Arabic version of the questionnaire is also available upon request. Please do not hesitate to contact the corresponding author for further assistance.
